# Comparison of 2 anesthetic protocols and surgical timing during cesarean section on neonatal vitality and umbilical cord blood parameters

**DOI:** 10.1186/s12917-023-03607-2

**Published:** 2023-02-13

**Authors:** Agnieszka Antończyk, Zdzisław Kiełbowicz, Wojciech Niżański, Małgorzata Ochota

**Affiliations:** 1 Faculty of Veterinary Medicine, Department and Clinic of Surgery, Wroclaw University of Environmental and Life Sciences, Pl. Grunwaldzki 51, 50-366 Wrocław, Poland; 2 Faculty of Veterinary Medicine, Department of Reproduction and Clinic of Farm Animals, Wroclaw University of Environmental and Life Sciences, Pl. Grunwaldzki 49, 50-366 Wrocław, Poland

**Keywords:** Cesarean section, Dog, Induction to delivery time, Propofol

## Abstract

**Background:**

The objective of this study was to evaluate the relationship between the mode of anesthesia, the time form the induction to the extraction of a puppy and the immediate postnatal vitality and umbilical cord blood gases parameters in cesarean section derived-puppies. Two different anesthetic protocols were used: inhalation using isoflurane (ISO) and combined—inhalation and epidural (EPI) with propofol being the induction agent.

**Results:**

Significant differences were found in ISO group in pH values, pCO2 levels and Apgar scores between puppies at different extraction times (< 30 vs. ≥ 30 min). In ISO group puppies extracted later were more acidic (7.16 vs. 7.22), had higher levels of pCO2 (69 vs. 57 mmHg) and lower Apgar scores at birth (1.2 vs. 2.5). On the contrary, in EPI group no differences were observed between the delivery time, umbilical blood gas parameters and puppies’ vitality. Furthermore, the dams from the EPI group required lower concentrations of isoflurane (MAC 1.11 ± 0.19 vs.1.37 ± 0.16, *p* < 0.001).

**Conclusions:**

Multiple pregnancies frequent in dogs lead to significant differences in extraction times between the first and the last puppy during cesarean section. Obtained results showed that the mode of anesthesia and the surgical time would influence the neonatal outcome during cesarean section in dogs. The higher concentration of isoflurane with the longer time of exposure had a negative effect on the initial newborn vitality as well as the umbilical cord blood gas parameters. Therefore, when performing CS in giant dog breeds or expecting many puppies in the litter, it is worth considering epidural component that allow for lower concentrations of inhalant agents, which may contribute to a better clinical condition of newborns.

## Background

One of the most popular anesthetic protocols for cesarean section (CS) in small animals practice are the inhalant anesthesia alone or supplemented with the neuroaxial block [1–6]. Many reports are available that show the superiority of combined anesthesia (inhalant plus neuroaxial blocks) over solely inhalant or total intravenous anesthesia (TIVA). However, any inhalant protocol requires an induction agent to facilitate intubation for the further administration of anesthetic gases. In veterinary medicine, one of the most widely used medication for induction is the intravenous bolus of propofol [1, 5–7].

It has been proven that the factor that greatly influences the CS outcome is the length of time the fetuses remain under anesthesia i.e. the time from induction of general anesthesia until newborns’ extraction from uterus (induction-delivery time—IDT). Most of authors agree that CS should be performed in timely fashion and advise dam’s clipping and scrubbing before the anesthetic agent administration to shorten to an absolute minimum the time between anesthesia induction and puppies’ retrieval, reducing the potential adverse effects of the anesthesia on fetuses [8–10]. However, there are some contradictory reports suggesting the benefits of time lapse between the general anesthesia induction and newborn delivery time during CS [2, 11, 12]. These opinions assume that a certain amount of time is needed for the maternal clearance of the administered induction agent. Hence, they suggest that a deliberate delay between the induction and the puppy extraction might subsequently lower the adverse effects of induction agent in the newborn.

It has been shown that after a single bolus of propofol it exhibits an immediate plasma concentration peak, followed by a rapid decline [11, 13]. In women [10], ewes [14] and bitches [2] propofol also readily crosses the placenta, reaching the fetal circulation rapidly, but its elimination from intrauterine blood stream is slower than from the maternal circulation. In children it has been presented that after a single intravenous administration of 2.5 mg/kg propofol in the mother, its concentration in the umbilical artery reached 1.75 µg/ml and 1.25 µg/ml after 5 and 7 min, respectively [12], which is higher than the mean concentration leading to the unconsciousness in children (973 ng/ml) [15]. Therefore, when performing CS, it seems justified to aim at lowering the dose of propofol (which may not be feasible) or lengthening the time between the induction and delivery of the newborn, in order to allow the concentration of circulating propofol to be reduced to a less depressive level for the newborn. Several other studies have also confirmed that in women the concentration of propofol and its potential adverse effects on newborns at delivery are dose-dependent and are also dependent on the time schedule from the induction and delivery [10, 16, 17]. Whereas Short and Bufalardi [11] recommended using 18–20 min time interval between propofol induction and delivery to reduce the respiratory depression in puppies and kittens. Finally, Groppetti et al. [2] found that in dogs maternal propofol concentration decreased 20 min after the induction and the Apgar scores increased with the extraction time, starting from 30 min after induction. Taking into consideration the above-mentioned reports, it was decided to divide the investigated puppies with regard to the removal time, into rapidly (under 30 min.) and slowly (over 30 min.) removed from the uterus. However, during CS, not only the induction agent would have consequences on the newborn outcome. The drugs used for general anaesthesia would also have an effect on puppies vitality. Inhalation anesthesia is considered reliable and secure, but commonly used gases would have an impact on the fetal neurological system. Wang et al. [18] reported a concertation dependent effect of isoflurane on developing fetal rat brains, however, they also mentioned species-specific differences in brain tissue vulnerability to isoflurane. Therefore, the second interest in our study was the comparison between the concentration of volatile agents during CS and the subsequent neonate outcome.

Veterinary reproduction and obstetrics in small animals, especially dogs, should also take into account that pregnancies are often multiparous and there is significant variation in size of individuals depending on breed, and therefore the number of pups per litter leads to notable differences in cesarean section and thus the time required between induction and the delivery of the last pup.

As presented above the available literature documents very divergent views on this matter, so it seemed warranted to conduct further research to clarify whether the lapse of time between induction and retrieval of puppies during CS will affect the condition of the newborns. To the best authors’ knowledge, there are no publications reporting the duration of anaesthesia on the umbilical cord blood gasses analysis and neonatal vitality in dogs. Therefore, the aim of the study was to assess the relationship between the time from the induction of general anesthesia to the extraction of a puppy, the subsequent vitality of a newborn and the umbilical cord blood parameters when using the two common anesthetic protocols for the cesarean section in dogs.

## Results

Twenty-five CSs were conducted under inhalant anesthesia (group ISO) and twenty-two under inhalant plus epidural (group EPI) protocol. The general data i.e. dams’ age, number of pups per litter and preoperative physiological parameters did not differ among the investigated groups (Table 1). The only differences were noted in the body weight (the bitches from ISO group weighted more) and the mean duration of anesthesia that was longer also in the ISO group.

**Table 1:** General clinical data and preoperative parameters (mean, SD) in bitches undergoing cesarean section maintained solely on isoflurane (group ISO) or with the addition of the epidural (group EPI)

Dams – general data	ISO	EPI
Parameter	Mean, SD	
No. of bitches	25	22
Age (years)	3.9 ± 1.8	4.1 ± 1.8
Body weight (kg)	**31.1** ± **21.9**^1^	**14.8 ± 10.3**
Pups investigated in study	74	76
Pups per litter^a^	4.7 ± 2.7	5.3 ± 2.8
Preoperative		
HR (bpm)	129.5 ± 22.4	116.8 ± 24.0
RR (breaths/min)	56.8 ± 31.6	52.2 ± 28.5
T (°C)	38.0 ± 0.4	37.8 ± 0.5
Anesthesia duration (min)	**65.8 ± 13.6**^2^	**55.5 ± 13.6**
Induction-delivery time (min)	27.28 ± 7.9	25.59 ± 5.5
Propofol dose (mg/kg)	4.7 ± 1.3	5.4 ± 1.8

^1^ – inicates difference between groups, *p* = 0.002

^2^—inicates difference between groups, *p* = 0.01

^a^—includes all puppies born in investigated cesarean sections

The number of puppies delivered in both investigated groups did not differ (74 vs. 76) and in both groups the stillborn puppies and those which failed umbilical cord blood collection were excluded from the study.

The dams’ intraoperative physiological parameters are shown in Table 2. Dams who received epidural component required lower isoflurane concentration to maintain the surgical plane of anesthesia (MAC was at 1.37 in ISO group vs 1.11 in EPI group, *p *< 0.001). Dams from both groups had episodes of hypotension (77.2% in EPI and 20% in ISO group) requiring rescue fluid administration, however, in the EPI group females expressed a significantly lower average systolic, diastolic and mean blood pressure (SBP, DBP, MBP respectively). In addition, differences were found in etCO2 levels between ISO and EPI group, but in both investigated groups the values remained within the normal range. All bitches were breathing spontaneously.

**Table 2:** Intraoperative variables in bitches undergoing cesarean section maintained solely on isoflurane (group ISO) or with the addition of the epidural block (group EPI) (mean, SD)

Parameter/Group	ISO	EPI
HR (bpm)	116.05 ± 13.66	114.97 ± 17.44
RR (breaths/min)	23.18 ± 9.84	28.98 ± 12.16
etCO2 (mmHg)	**43.37 ± 5.73**^1^	**38.37 ± 7.46**
SpO2 (%)	98.41 ± 1.77	97.43 ± 3.37
SBP (mmHg)	**104.61 ± 20.36**^2^	**87.19 ± 13.14**
DBP (mmHg)	**52.67 ± 14.75**^3^	**39.71 ± 13.03**
MBP (mmHg)	**70.92 ± 17.01**^3^	**56.54 ± 13.05**
MAC	**1.37 ± 0.16**^4^	**1.11 ± 0.19**

^1^—inicates difference between groups, *p* = 0.01

^2^—inicates difference between groups, *p* = 0.001

^3^—inicates difference between groups, *p* = 0.003

^4^—inicates difference between groups, *p* < 0.001

**Fig. 1:**
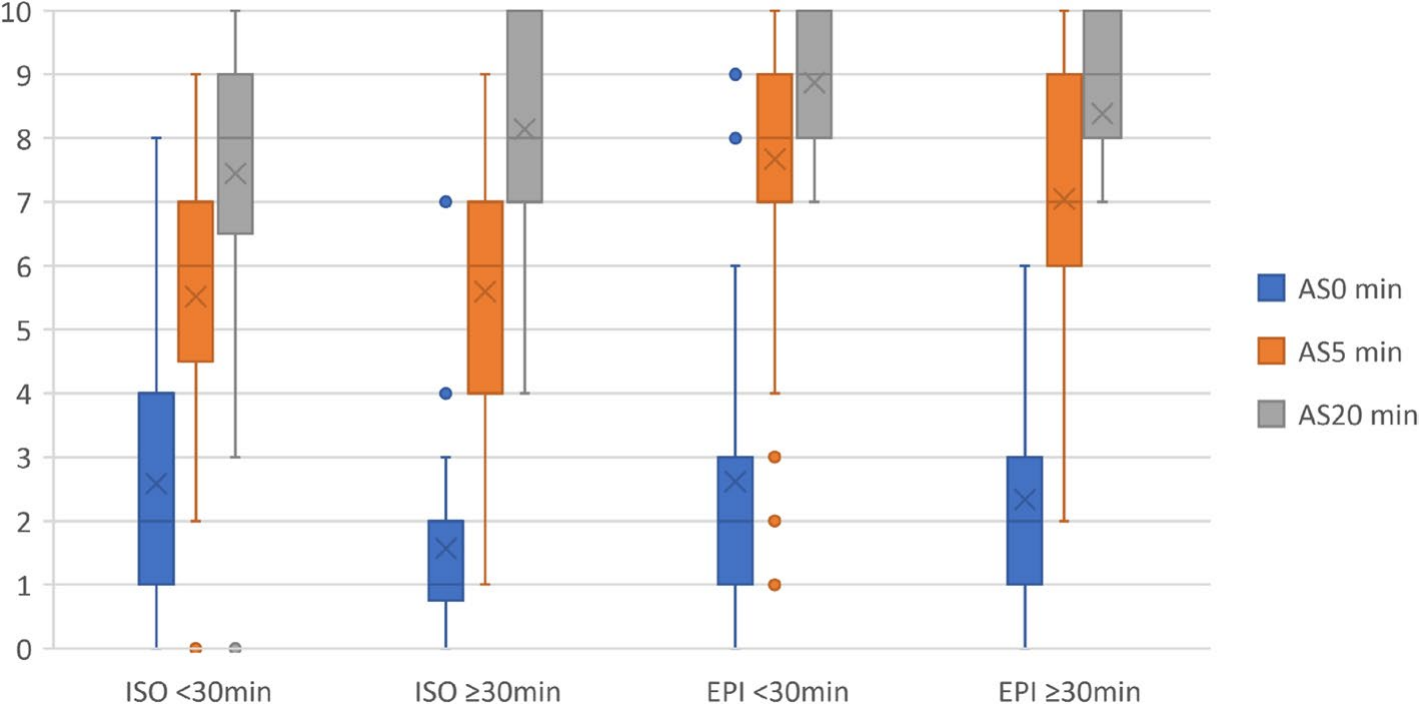
Box plot of puppies’ vitality scores (at 0, 5 and 20 min.) evaluated in newborns with delivery time (IDT) less (IDT < 30 min) or more (IDT ≥ 30 min) in ISO and EPI group. ISO – dams maintained solely on isoflurane. EPI – dams maintained on isoflurane with the addition of the epidural block

The comparison of the vitality and umbilical blood gas analysis in puppies from IDT < 30 and IDT ≥ 30 groups is presented in Table 3. Puppies extracted under 30 min after induction (IDT < 30 group) were less acidic (pH at 7.22 ± 0.06 in IDT < 30 group vs. 7.16 ± 0.08 in IDT > 30 group; *p* = 0.02). Moreover, puppies from IDT ≥ 30 group and from dams in ISO group had the highest pCO2 (69.09 ± 11.43 mmHg) of all the investigated neonates. The Apgar scoring showed also the effect of the induction—delivery time (IDT) on the newborns’ vitality and health status with the lowest scores found in those newborns that were delivered later (IDT ≥ 30), or those from the ISO group (Fig. 1). The analysis of the results showed that puppies in ISO group that remained in uterus longer (≥ 30 min.) had significantly worse condition at the first Apgar assessment (AS at birth 1.21 in IDT ≥ 30 vs. 2.51 in IDT < 30 group, *p* = 0.001). Interestingly, similar differences were not found in puppies from EPI group, here regardless the IDT the initial puppies’ vitality was the same. In the following evaluations, the IDT effect was not significant in both groups. However, newborns born under solely ISO anesthesia were scored lower in all assessments, regardless the delivery time (Fig. 1).

**Table 3:** Umbilical blood gas analysis in puppies with shorter (IDT < 30 min) and longer (IDT ≥ 30 min) delivery time after propofol bolus in ISO and EPI group. Differences between both groups were assessed using the Kruskal–Wallis test

Parameter/Group	ISO		EPI	
IDT	IDT < 30 min	IDT ≥ 30 min	IDT < 30 min	IDT ≥ 30 min
n	44	30	55	21
pH (units)	**7.22 ± 0.06**^b^ **(7.01–7.31)**	**7.16 ± 0.08** **(7.12–7.28)**	7.20 ± 0.08 (7.00–7.36)	7.19 ± 0.07 (7.00–7.32)
pCO2 (mmHg)	**57.23 ± 11.34**^b^ **(41.10–84.10)**	**69.09 ± 11.43**^a^ **(51.70–95.80)**	60.31 ± 13.88 (30.20–90.4)	**61.02 ± 14.84**^a^ **(32.1–91.7)**
HCO^3^ (mmol/L)	23.42 ± 3.11 (17.00–29.70)	24.77 ± 3.10 (22.6–31.00)	23.37 ± 3.51 (16.4–32)	23.25 ± 4.32 (16.00–31.50)
BE (b) (mmol/L)	-5.00 ± 2.73 (-11.20–0.40)	-5.27 ± 3.68 (-5.00 – -1.50)	-5.36 ± 4.05 (-14.00 – 10.00)	-5.75 ± 3.78 (-12.10 – 0.50)
Lac (mmol/L)	2.85 ± 1.39 (0.94 ± 9.33)	2.45 ± 0.79 (2.18–4.69)	2.63 ± 1.13 (0.76–5.28)	2.62 ± 1.55 (0.57–7.32)
Glu (mg/dl)	72.09 ± 22.24 (18.00–114.00)	80.53 ± 19.71 (51.00–131.00)	72.78 ± 24.53 (10.00–138.00)	84.00 ± 24.17 (39–140)

*IDT* induction—delivery time, *ISO* dams maintained solely on isoflurane, *EPI* dams maintained on isoflurane with the addition of the epidural block

^a^the same superscript denotes differences between groups

^b^difference within the group

## Discussion

This study presents for the first time the relationship between the time from the induction of general anesthesia to the extraction of a puppy and the subsequent vitality and metabolic status of a newborn. Furthermore, it compares two different anesthetic protocols, routinely used for the cesarean section in dogs, in terms of puppy’s immediate postnatal outcome and the parameters of umbilical cord blood, in order to determine the optimal surgical time during elective CS in dogs to maintain the well-being and vitality of the newborns.

Our results showed that the maternal preoperative parameters were comparable in both groups of investigated bitches. Similarly, the dose of propofol which all bitches required for induction was the same in both groups (4.7 mg/kg vs. 5.4 mg/kg, *p* = 0.11 in ISO and EPI group, respectively). Nevertheless, some intraoperative parameters differed between groups. Dams from ISO group required a higher concentration of isoflurane whereas mothers from EPI had significantly lower blood pressure (BP). Hypotension is a common and widely reported side effect of neuroaxial block [1, 3, 19–22] that follows the spinal block-induced sympatholysis and contributes to vasodilation. Moreover, it has been reported that short episodes of maternal hypotension before delivery did not have a significant impact on newborns’ postnatal condition [4, 21, 23]. On the contrary, the isoflurane consumption during CS may expose fetuses a high concentration of volatile agents that may affects their central nervous system and thus the subsequent neonatal vitality and the cesarean section outcome [18].

In the presented study, the umbilical cord blood gases (UCBGA) results and initial Apgar scores were found to be significantly different only in newborns from the ISO group who remained in the uterus longer (IDT > 30 min). These puppies were more acidic (had lower pH of 7.16 ± 0.08) and had hypercapnia (higher pCO2 69.09 ± 11.43), when compared to the puppies from the ISO group which were delivered quicker (IDT < 30 min.; pH: 7.22 ± 0.06 and pCO2: 57.23 ± 11.34 mmHg, respectively). The observed hypercapnia would reflect the perinatal asphyxia developed during surgery and may indicate difficulties in gas exchange across placenta. In addition, the acidosis and hypercapnia more likely worsened the general condition of ISO group puppies at birth. The ISO newborns delivered 30 min and later, received on average only 1.21 points in Apgar score, which was significantly lower than the rest of the examined neonates (AS of ISO puppies delivered < 30 min. was: 2.51, whereas AS in EPI group was 2.33–2.62 points). In newborns born to dams under epidural anesthesia, the effect of longer intervals between the propofol induction and delivery of each puppy (IDT) was not detected for either UCBGA or for the Apgar score at any of the time point examined. Indeed, the vitality and Apgar scores of puppies extracted later (IDT ≥ 30 min.) in the EPI group were not worse than in newborns from the same group but delivered under 30 min (IDT < 30 min.).

The comprehensive evaluation of the obtained results is difficult because reports concerning the UCBGA evaluation in dogs are not available. The attempts to find a relationship between the results presented in our study and the findings obtained in human medicine is not only difficult, but in true not appropriate due to the huge differences in pregnancy/delivery physiology, mainly the incomparable IDT regimen used during CS in a singleton human pregnancy. Hu et al. [24] have detected no differences in UCBGA in human babies between the short and long induction—delivery times, but the mean recorded IDT values were 6.9 ± 1.2 and 18.0 ± 1.9 min., respectively. Whereas, Daillon et al. [16] have recorded the UCBGA within the normal range for human newborns with the mean IDT time of 25.9 and 20.2 min. Finally, Maayan-Metzger et al. [17] concluded that the duration of the interval between the induction and delivery did not have a significant effect on any of the measured neonatal parameters. However, one has to remember that the average time frames during CS in humans were significantly shorter than similar times recorded for dogs in our study. In our research, IDT ranged from 10 to 44 min, the shortest was noted in a single-pup miniature breed (Yorkshire terrier) pregnancy, while the longest induction do delivery time was registered for the last pup in giant breed bitch (Great Dane) with a litter of 12 puppies. Hence, attempts to compare or identify a relationship between our research and human reports are unfortunately counterproductive and seem unjustified.

The published in human medicine, rather short and comparable among different reports, time frames from the induction to delivery could also be the explanation for the lack of the IDT effect on human babies’ vitality and Apgar scoring [10, 16, 24]. In dogs, however, longer IDTs, are inevitable in multifetal pregnancies typical for canine reproduction, and may lead to the differences in immediate postnatal condition of puppies described here. In one study published by Groppetti et al. [2] there were reported higher Apgar scores with the increasing time frame from the induction to delivery, and the clear differences were observed when the IDT was above 30 min after induction. These authors concluded that that the improvement in puppies’ condition with the longer time from the induction to the extraction from the uterus, resulted from the maternal metabolism and a decrease in serum propofol concentration, observed 20 min. after induction. However, in that study, the CSs were performed using alfa-2 agonist (dexmedetomidine) and propofol for the induction, which had an undeniable impact on the subsequent condition of the newborns. Our findings partially contradict that assumption, mainly for the CS maintained solely under the isoflurane anaesthesia (ISO group). We found that in ISO group puppies extracted later were not better, but even worse, obtaining a significantly lower Apgar scores and being more acidic compared to those delivered earlier (under 30 min). Whereas neonates from EPI group, regardless the delivery time (before or after 30 min form the induction with propofol) had similar Apgar scores and UCBGA parameters. In our studies the propofol bolus for the induction of anesthesia seemed to be insignificant for the subsequent neonatal vitality. Our dams were clipped and scrubbed after the propofol administration and the shortest induction–delivery time was 10 or 14 min (for ISO and EPI group respectively), so if relevant, we should have noticed the negative impact of the circulating propofol in the earlier born puppies. However, no such relationship was found, and the obtained results suggest that the condition of newborns was rather related to the type and duration of anesthesia. The prolonged exposure to the higher concentration of inhalant agent (isoflurane) during CS, had evident adverse effect on vitality and cord blood parameters in puppies. In the above-mentioned paper of Groppetti et al. [2] the volatile agent concentration or the detailed intraoperative parameters that could have a significant impact on newborns condition were not reported. The lack of published data in this field makes it very difficult to compare and discuss our findings with the available literature. Based on our results we could assume that the epidural component during CS should be recommended as beneficial for the condition of newborn puppies.

The available literature on maternal and neonatal effects of neuroaxial and general anesthesia during CS is extensive and not always conclusive. However, most studies reported the superiority of newborn outcome in elective CS performed under regional versus general anesthesia, both in humans [25–30] and dogs [1, 4, 5]. From the available literature it can be concluded that isoflurane can lead to dose and time dependent damage various tissues [31–33]. Moreover, in a published study on rats, Wang et al. [18] proved that the prenatal fetal exposure to high (3%) but not low (1.3%) isoflurane concentration induced neurodegeneration in growing brains of developing in utero fetuses. It can therefore be assumed that the severity of acidosis and the decrease in Apgar score in the ISO puppies delivered 30 min and later after the induction were the result of prolonged prenatal exposure to a higher concentration of isoflurane.

In the presented study a single dose of meloxicam was used in pregnant dams. Many CS anesthetic protocols recommend administering NSAID after the last puppy removal, as prenatal exposure to these medications can affect the brain, kidney, lungs, skeleton, gastrointestinal tract, and cardiovascular system of the fetus [34]. However, in human medicine papers presenting different approaches can also be found [35–37]. Their authors indicate that perioperative NSAID administration reduces pain scores, analgesic consumption, and improves recovery and quality of breastfeeding. The authors’ personal observations also confirm that the lack of a perioperative NSAID component during CS results in poorer patient anesthetic compliance, increased anesthetic consumption and the necessity of opioid administration before the removal of puppies, which affects the postnatal outcome of the newborn.

## Conclusions

Multiple pregnancies frequent in dogs lead to significant differences in extraction times between the first and the last puppy during cesarean section. Presented results showed that prolonged fetal exposure to higher (1.37 vol.%) concentration of isoflurane worsened the condition of newborns and negatively affected the cord blood gas parameters. Whereas, with lower (1.1 vol.%) isoflurane concentrations the puppies’ vitality and cord blood gasometry did not differ regardless of time frame from induction to delivery. Moreover, the length of time between the administration of induction agent and delivery seemed insignificant for the initial puppy’s outcome.. Therefore, when performing CS in giant dog breeds or expecting many puppies in the litter, it is worth considering epidural component that allow for lower concentrations of inhalant agents, which may contribute to a better clinical condition of newborns.

## Materials and methods

### Animals

Forty seven client-owned bitches belonging to 18 breeds and scheduled for the elective cesarean section were enrolled in this study. The study protocol was approved by the II Local Ethics Committee for Animal Experiments (No 047/2020). All methods were carried out in accordance with relevant guidelines and regulations, and the manuscript adheres to ARRIVE guidelines. Data was collected between May 2020 and February 2022. Qualified bitches were deemed healthy based on physical examination findings performed by the authors and bloodwork (complete blood count and basic biochemical profile). The age of the bitches ranged from 1.5 to 8 years (mean 4.04 ± 1.76 years), whereas the body weight (BW) from 3 to 77 kg (mean 23.50 ± 19.13). In every case the cesarean section was planned independently of this research. Solely the clinical indications (e.g., very numerous or single pup pregnancies), dystocia in previous history or breed related factors (brachycephalics) were considered when scheduling the patient for the CS. The elective cesarean was performed on 63–65 day after the LH peak and after a full clinical examination and pregnancy scan to evaluate fetal heart rate and gastrointestinal motility. Based on the obtained results, the CS was performed or postponed to the following day.

### Allocation to the study groups

The date for the elective cesarean section was identified based on the progesterone levels measured during heat and followed by LH peak determination and was performed on 63—65 day after the LH peak. All bitches underwent a full clinical examination before qualification for the elective CS. The pregnancy was monitored weekly (ultrasonography) from the day of its confirmation to the scheduled delivery date. On the 63rd day from LH peak, each dam underwent a thorough obstetrical examination, the presence of milk was checked, and a detailed pregnancy ultrasound examination was performed to assess fetal heart rate and gastrointestinal motility. Based on the obtained results, the surgery performed on the given day or postponed to the following day, in cases where no clinical symptoms of first stage of labour were observed, no signs of fetal distress, and vague or no peristalsis were visible on ultrasonography.

### Anesthetic protocol

The dams were assigned in one of two standard anesthetic protocols including inhalant (ISO) or balanced (inhalant plus epidural, EPI) anesthesia. The decision on the type of anesthesia used in each case was made by the anesthesiologist and surgeon responsible for the procedure and independently of this study. Regardless the group allocation, no premedication was used, but at least 30 min before the surgical procedure all bitches received a single dose of meloxicam (0.2 mg/kg s.c., Metacam 5 mg/ml, Boehringer Ingelheim, Poland). The general anesthesia was induced with propofol (Propofol-Lipuro®, 10 mg/ml B. Braun Melsungen AG, Germany) at initial dose of 1 mg/kg to effect, in order to permit the tracheal intubation. The anesthesia was maintained with isoflurane (IsoVet®, Piramal Healthcare, United Kingdom) in oxygen. In the EPI group, lidocaine (Lignocainum Hydrochloricum WZF 2%, Polfa Warszawa, Poland) was administered into epidural space at a dose of 3–4 mg/kg immediately after induction with propofol, at the same time when the endotracheal tube was fixed and inflated to match the timing of the procedure in the ISO group. To perform epidural, the dam was placed in sternal recumbency and the epidural injection was performed into the lumbosacral space. The entry of the needle into the epidural space was confirmed by the presence of a distinct ‘popping sensation’ as a result of penetrating the ligamentum flavum, hanging drop technique and the lack of resistance to injection. Anal sphincter relaxation was taken as the confirmation of the correct epidural lidocain administration. In the ISO group, analgesia was continued with methadone (Comfortan® Eurovet Animal Health BV, Holand) or buprenorphine (Bupaq Multidose® Richter Pharma AG, Austria), after the last puppy removal. Cesarean sections were carried out with a standard ventral midline approach, which is a routine protocol as described before [4, 38].

Throughout the surgical procedure, vital parameters of the dams (heart rate, respiratory rate, end-tidal CO2, blood pressure, oxygen saturation, temperature) were monitored constantly (Datex-Ohmeda S5 monitor Helsinki, Finland) and noted in 2 min intervals. Blood pressure was measured with a non-invasive, oscillometric method with the proper size of a cuff placed on the forearm. Minimal alveolar concentration (MAC) of isoflurane, provided by the Datex-Ohmeda S5 monitor, was also investigated.

All bitches were administered intravenous fluids (crystalloids) at the rate of 5 ml/kg/h. The IV bolus of fluid (crystalloid or colloid at 10–20 ml/kg or 3–5 ml/kg, respectively) was given when the hypotension, defined as mean arterial pressure below 60 mmHg, occurred.

Anesthesia time was defined as total time from induction of general anesthesia to the extubation. The time from propofol administration to each puppy extraction was also measured (Induction-Delivery Time, IDT).

### Umbilical Cord Blood Gas Analysis (UCBGA)

The blood samples for gas analysis were collected in heparinized syringes and analyzed immediately after sampling using EPOC VET handheld analyzer (Siemens Healthineers, Germany) which is a point-of-care diagnostic device certified for veterinary use*.* A minimum of 100 mcl of mixed umbilical cord blood was needed to conduct the test. The umbilical blood sample was taken immediately after the removal of fetal membranes from the puppy’s head but before placenta detachment. The umbilical cord was double clamped with the first forceps placed close to the placenta and the second about 1 cm from the fetal abdominal wall, then blood was collected.

### Apgar Scoring (AS)

The initial newborn evaluation was carried out by the experienced member of staff using modified Apgar scoring system [6]. The first scoring was done before any neonatal assistance was started (0 min), and then at 5 and 20 min. Finally, the last clinical examination of each newborn was carried out before discharge. The Apgar evaluation was performed according to the following scoring: heart rate (HR): > 220 bpm (2 points), 180 – 220 bpm (1 point) to < 180 (0 points); respiration rate (RR): > 15 breaths per minute (2 points), 6—15 (1 point) to < 6 (0 points); the irritability reflex detected after a gentle compression of a tip of a paw—evaluated based on the degree of reaction: crying and quick leg retraction (2 points), weak leg retraction and no or just weak vocalization (1 point), and no leg retraction and no vocalization (0 points); spontaneous movement of a newborn: 2—strong movement, 1—weak movement, and 0 – absent; mucous membranes color: dark pink (2 points), light pink (1 point) and pale to cyanotic (0 points). The total points received sum up for the final Apgar score: 7—10, no distress, healthy newborns; 4—6, moderate distress, weak newborn and 0—3, severe distress, critical newborns [6].

### Study design

Pregnant dogs were assigned to experimental groups based on the anesthetic protocol used for CS and the study was planned as a controlled clinical, non-blinded study. Investigated bitches were divided into two groups: ISO group (anesthesia maintained solely with isoflurane) or EPI group (anesthesia maintained with isoflurane plus epidural block) and puppies were divided retrospectively according to the time that elapsed between the moment of induction and moment of delivery of each individual puppy (Induction—Delivery Time: IDT). Two groups of puppies were established: Group IDT < 30—newborns delivered below 30 min and Group IDT ≥ 30—newborns delivered 30 min or later after the induction of anesthesia. All puppies were extracted from uterus as quickly as possible after induction, and the induction—delivery time depended mainly on the size of the bitch and the number of pups per litter, so in some cases puppies from the same litter were classified into different groups depending on the time of their retrieval from the uterus (IDT < 30: under or IDT ≥ 30: over 30 min. from the induction). The time cut-off value was set at 30 min according to the results obtained by Groppetti et al. [2]. Vitality of each pup (modified Apgar score) was evaluated immediately after birth (0 min – to assess pup condition before any neonatal assistance was instituted), and then 5 and 20 min after the first evaluation.

### Statistical analysis

Statistical analysis included descriptive statistics and normality testing using the Kolmogorov–Smirnov test with the Lilliefors correction. When the results did not follow normal distribution non-parametric tests were applied. Pre- and intraoperative parameters of the dams were compared between groups using U-Mann-Withney test. Puppies in each study group were divided based on induction-delivery time and Apgar scores and umbilical blood gas parameters were compared between the 4 groups with non-parametric Kruskal–Wallis test.

## Data Availability

The datasets used and/or analysed during the current study available from the corresponding author on reasonable request.
